# The monitoring and use of Pro re nata (PRN) psychotropic medication for people with learning disabilities on an inpatient ward

**DOI:** 10.1192/bjo.2021.278

**Published:** 2021-06-18

**Authors:** Heena Mistry

**Affiliations:** Leeds and York Partnership NHS Trust

## Abstract

**Aims:**

This project was designed to evaluate the use of PRN medication and PRN monitoring charts on an adult learning disability ward. These charts had been designed by the trust to provide us with a way of monitoring the use of psychotropic PRN medication to ensure monitoring of treatment response, physical health and side effects.

**Method:**

The data were collected from PRN monitoring charts, electronic case notes and electronic prescribing chart records for all patients on an adult learning disability inpatient unit. The sample consisted of 7 patients who had been prescribed and/or received PRN psychotropic medication over a five week period. Quantitative data were derived by simple calculation for the total amount of PRN medication used and number of PRN monitoring charts completed. Qualitative data were collected of prescription charts and PRN protocols which is supposed to guide treatment.

**Result:**

Out of all the incidences where PRN medication was administered, only 64% of monitoring charts were completed. Out of the 7 patients on the ward, 6 had PRN protocol charts and for only 5 patients these were followed.

**Conclusion:**

Clinical practice must be improved. The results were presented to ward staff and doctors to discuss the implications for patient care and ways to improve clinical practice by ensuring full monitoring of the use of PRN medication to help reduce the overmedication of people with learning disability by improving the use of the PRN charts. NICE guidelines and The Royal College of Psychiatrists have published guidelines on the prescription of psychotropic drugs for people with learning disabilities. NHS England have also published an article to discourage over-medication of people with learning disabilities. There is a risk that doctors are prescribing medication to treat behaviour that is an expression of distress or a mode of communication rather than a mental disorder. Doctors have a responsibility to ensure they have fully assessed the person's potential to benefit from medication before they prescribe. The audit would serve to provide a baseline for this team prior to any audits in the future.

